# The *LITAF*/*SIMPLE* I92V sequence variant results in an earlier age of onset of CMT1A/HNPP diseases

**DOI:** 10.1007/s10048-014-0426-9

**Published:** 2014-10-24

**Authors:** Elena Sinkiewicz-Darol, Andressa Ferreira Lacerda, Anna Kostera-Pruszczyk, Anna Potulska-Chromik, Beata Sokołowska, Dagmara Kabzińska, Craig R. Brunetti, Irena Hausmanowa-Petrusewicz, Andrzej Kochański

**Affiliations:** 1 Neuromuscular Unit, Mossakowski Medical Research Centre, Polish Academy of Sciences, Pawinskiego 5, 02-106 Warsaw, Poland; 2 Biology Department, Trent University, Peterborough, ON K9H 7B8 Canada; 3 Department of Neurology, Medical University of Warsaw, Warsaw, Poland; 4 Bioinformatics Laboratory, Mossakowski Medical Research Centre, Polish Academy of Sciences, Warsaw, Poland

**Keywords:** LITAF, Charcot-Marie-Tooth disease type 1A, Gene dosage, Biomarkers

## Abstract

Charcot-Marie-Tooth disease type 1A (CMT1A) and hereditary neuropathy with liability to pressure palsies (HNPP) represent the most common heritable neuromuscular disorders. Molecular diagnostics of CMT1A/HNPP diseases confirm clinical diagnosis, but their value is limited to the clinical course and prognosis. However, no biomarkers of CMT1A/HNPP have been identified. We decided to explore if the *LITAF*/*SIMPLE* gene shared a functional link to the *PMP22* gene, whose duplication or deletion results in CMT1A and HNPP, respectively. By studying a large cohort of CMT1A/HNPP-affected patients, we found that the LITAF I92V sequence variant predisposes patients to an earlier age of onset of both the CMT1A and HNPP diseases. Using cell transfection experiments, we showed that the LITAF I92V sequence variant partially mislocalizes to the mitochondria in contrast to wild-type LITAF which localizes to the late endosome/lysosomes and is associated with a tendency for PMP22 to accumulate in the cells. Overall, this study shows that the I92V LITAF sequence variant would be a good candidate for a biomarker in the case of the CMT1A/HNPP disorders.

## Introduction

Charcot-Marie-tooth disease type 1A (CMT1A) and hereditary neuropathy with liability to pressure palsies (HNPP) belong to the group of most common heritable neuromuscular disorders caused by submicroscopic duplication or deletion of the 17p11.2-p12 region, respectively [14, 10, 3], Chance et al., 1993]. Clinical variability in CMT1A and HNPP is a well-known phenomenon [11, 15]. Indeed, marked variability is noted in monozygotic CMT twins when reference is made to clinical and electrophysiological features [4]. Presently, the genetic counseling of CMT1A/HNPP-affected patients is limited to the confirmation of clinical diagnosis and the estimation of the recurrence risk of these disorders. However, while clinical variability is a well-known phenomenon in the case of both CMT1A and HNPP, no biological basis for it is known. Thus, while the CMT1A/HNPP diagnosis may be precise, molecular genetics do not seem to help in issues relating to the prognosis or clinical course of these disorders.

It has been suggested that lipopolysaccharide-induced tumor necrosis factor-alpha factor (LITAF) may play a significant role in CMT. While patients who initially lacked mutations to known genes were designated to the CMT1C subtype, the molecular basis of the latter has recently been identified as a mutation in LITAF [21]. Such LITAF mutations associated with CMT, such as I92V, occur mostly in the C-terminus of LITAF. The mechanism by which mutations in LITAF give rise to the CMT1C disease phenotype remains unknown. Although mutations in LITAF that cause CMT1C have been shown to result in mislocalization of LITAF from the endosome/lysosome to the mitochondria (Ferreira [7]), and these mutations result in defects in regulating endosomal trafficking and signaling [9, 23].

The dosage of the peripheral myelin protein 22 (*PMP22*) gene seems to be a critical factor where the clinical course of CMT1A/HNPP is concerned. Thus, the number of copies of the *PMP22* gene has been shown to correspond with the clinical course of neuropathy in laboratory transgenic animals [18, 22]. Moreover, an experimental approach entailing the reduced expression of the *PMP22* gene resulted in an improved clinical course of CMT in transgenic animals (mice/rats) [19]. Given the potential role of *PMP22* regulatory sequences in the gene-dosage effect, we decided to analyze an extended region of the 5′UTR sequence encompassing 5000 kb and the coding sequence of the *PMP22* gene in patients with CMT1A/HNPP (duplication/deletion of the *PMP22* gene). Unfortunately, we failed to detect any significant variants within the *PMP22* regulatory sequence that could be responsible for the gene-dosage effect and the observed clinical variability in the CMT1A/HNPP diseases [20].

## Materials and methods

### Reagents, cell lines, and antibodies

Baby green monkey kidney (BGMK) was obtained from the American Type Culture Collection (ATCC; Manassas, VA). BGMK cells were cultured in Dulbecco-modified Eagle’s medium (DMEM; HyClone, Ottawa, ON) with 7 % fetal bovine serum (FBS; HyClone), 2-mM L-glutamine, 100-U/mL penicillin, and 100-μg/mL streptomycin, at 37 °C with 5 % CO2. The following antibodies/probes were used during immunofluorescence: Lysotracker®DND-99 from Molecular Probes (Burlington, ON), the 9E10 mouse and mouse myc monoclonal antibody from Roche (dilution 1/100; Indianapolis, IN), rabbit anti-FLAG (dilution 1/100; Burlington, ON), and FITC/Cy3-conjugated goat anti-mouse or anti-rabbit immunoglobulin G (IgG) from Jackson ImmunoResearch Inc. (dilutions 1/100, 1/200 respectively; West Grove, PA, anti-CD63 antibody from Invitrogen (dilution 1/100; Burlington, ON); and MitoTRACKER Red FM from Molecular Probes (Burlington, ON). LITAF WT was synthesized by GenScript (Piscataway, NJ). Myc-tag was added to the N-terminus of the protein to facilitate imaging. PMP22 was synthesized by Sino Biological Inc. (BC019040, Beijing). The gene was pCMV/hygro and contained a flag-tag.

### Patients and genetic analysis

One hundred six patients affected with CMT were examined by neurologists at the Neuromuscular Unit and Warsaw Department of Neurology. Family trees of at least three generations were constructed for all the patients. The clinical diagnosis of CMT/HNPP was then confirmed in all patients by means of electromyography examination (EMG). Patients displaying symmetrical, generalized neuropathy involving upper and lower limbs were included in this study. All members of the CMT1A/HNPP-affected families gave their signed informed consent for the study, which gained the approval of the local Ethics Committee at Warsaw Medical University (approval No. 120/2008.). The control group consists of 50 unaffected individuals. Genomic DNA was extracted from peripheral blood lymphocytes using the salting-out procedure. The duplication/deletion of the *PMP22* gene was confirmed using Real-Time PCR (Q-PCR) [1]. This was performed in the context of a multiplex assay making use of two TaqMan probes labeled with FAM (*PMP22* gene) and VIC (human serum albumin gene). The Q-PCR reaction was performed on the ABI-7500 Real-Time PCR system (Applied Biosystems). The relative dosage (RQ) of the *PMP22* gene ranges from 0.700 to 1.090 in normal individuals (two copies of the *PMP22* gene), from 0.359 to 0.595 in HNPP patients (one copy of the *PMP22* gene), and from 1.176 to 2.324 in CMT1A patients (three copy of the *PMP22* gene) [5].

The three coding exons 2–4 of the *LITAF* gene were amplified by PCR (primer sequences previously described by [21]). The PCR products were sequenced directly using a BigDye^TM^ Terminator Version 1.1 Ready Reaction Cycle Sequencing kit on the ABI 3730/xl genetic analyzer (Applied Biosystems). The *LITAF* gene sequence was analyzed by comparison with reference sequence NM_004862.3 (transcript variant 1).

Total RNA was isolated from peripheral blood lymphocytes using the TRIzol Reagent, in line with the instructions from the manufacturer (Invitrogen).

RT-PCR reactions were carried out using a First Strand cDNA Synthesis Kit (Fermentas), again in line with the manufacturer’s instructions.

### Statistical analysis

The data was presented as means ± SD, medians, and ranges of values [Table 1]. Differences in values assumed by the variables were assessed using the Mann–Whitney *U* test. The *p* < 0.05 level was considered statistically significant, data being analyzed using the *StatSoft* statistical software package version 5.

**Table 1:** Correlation between CMT1A/HNPP age at onset (AOE) and I92V mutation within *LITAF* gene

Patients	Parameters	Age at onset (years)	*p* value
HNPP > 10 + I92V	mean ± SD (*n*)	18 ± 6 (16)	0023
	median	17.5	
	min-max	11–32	
HNPP > 10	mean ± SD (*n*)	37 ± 21 (7)	
	median	36	
	min-max	14–71	
CMT1A > 10 +I92V	mean ± SD (*n*)	23 ± 10 (15)	0005
	median	20	
	min-max	11–47	
CMT1A > 10	mean ± SD (*n*)	36 ± 14 (30)	
	median	35.5	
	min-max	13–67	
CMT1A < 10 + I92V	mean ± SD (*n*)	4.8 ± 3.2 (11)	0.963
	median	4	
	min-max	1.3–10	
CMT1A < 10	mean ± SD (*n*)	5.4 ± 4.4 (7)	
	median	3	
	min-max	1.5–10	

*HNPP* > *10* + *I92V* patients with HNPP with AOE after 10 year of life with sequence variant Ile92Val in *LITAF* gene, *HNPP* > *10* patients with HNPP with AOE after 10 year of life without sequence variant Ile92Val in *LITAF* gene, *CMT1A* > *10* + *I92V* patients with CMT1A with AOE after 10 year of life with sequence variant Ile92Val in *LITAF* gene, *CMT1A* > *10* patients with CMT1A with AOE after 10 year of life without sequence variant Ile92Val in *LITAF* gene, *CMT1A* < *10* + *I92V* patients with CMT1A with AOE before 10 year of life with sequence variant Ile92Val in *LITAF gene*, *CMT1A* < *10* patients with CMT1A with AOE before 10 year of life with sequence variant Ile92Val in *LITAF* gene

### PCR and expression plasmids

LITAF WT and the I92V LITAF mutation were amplified using a 50-μL PCR mixture containing 1× PCR buffer (Invitrogen), 3.0-mM MgCl2 (Invitrogen), 0.2-mM forward and reverse primers, and 2.5-U Taq DNA polymerase (5 U/μL; Invitrogen). LITAF WT and mutations were used as template DNA for the following primers: 5′-ATGGAGCAGAAACTGATTAGT-3′ (LITAF-forward), 5′-TACCAGTCT TTGTAAGTTCC-3′ (LITAF-reverse). The cycling conditions used were 94 °C for 30 s, 52 °C for 30 s, and 72 °C for 90 s for 30 cycles. The resulting PCR product was cloned into pTARGET (Promega, Madison).

### Sequencing

In order to confirm that transformation into cloning vectors was performed correctly, and to identify potential LITAF mutations, LITAF samples were prepared and sent for sequencing at the Robarts Institute (London, ON). Once sequencing was completed, data was analyzed using Codon Aligner 4.0.4 (Centerville, MA).

### Transfections

BGMK cells were grown to 70 % confluency and transfected using a polyethylenimine (PEI) reagent. DNA (5 μl) was diluted with 400 μl of serum-free DMEM. PEI was added in a 4:1 ratio of PEI:DNA and left to incubate at room temperature for 15 min. The transfection mix was then added to cells along with fresh DMEM.

### Immunofluorescence

In 24-h post-transfection, the cells were probed with MitoTRACKER Red FM or LysoTRACKER then fixed using 3.7 % paraformaldehyde solution in phosphate buffer solution (PBS) and permeabilized using a 0.1 % Triton X-100 solution in PBS. Cells were blocked for 2 h at room temperature in block buffer (5 % bovine serum albumin (BSA) (w/v), 50-mM Tris HCl (pH 7.4), 150-mM NaCl, 0.5 % NP-40 (v/v)) followed by several 5-min washes in wash buffer (1 % bovine serum albumin (BSA) (w/v), 50-mM Tris HCl (pH 7.4), 150-mM NaCl, 0.5 % NP- 40 (v/v)). Primary antibody diluted in wash buffer was incubated on cells for 1 h at room temperature. The primary antibody was removed following several 5-min washes in wash buffer, and secondary antibody was diluted in wash buffer and applied to cells. The secondary antibody was left on cells for 1 h at room temperature in darkness before removal with several 5-min washes in wash buffer. Cells were mounted using VECTASHIELD (Vector Labs, Burlington, ON), and fluorescence was detected using a Leica DM SP2 confocal microscope (Leica, Wetzlar, Germany). Images were assembled using Adobe Photoshop CS4 (Adobe, San Jose, CA). This process was repeated at least three times, and results were analyzed using ImageJ by using the method described by Burgess et al. [2].

## Results

### The I92V LITAF sequence variant predisposes patients to an earlier age of onset of CMT1A and HNPP diseases

We found that the presence of I92V heterozygous substitution in the *LITAF* gene is associated with an earlier age at onset of CMT1A and HNPP diseases in the group of patients manifesting with CMT1A/HNPP after 10 years of age. In this group of patients, we found a statistically significant association (*p* = 0.005 and *p* = 0.023) for CMT1A and HNPP, respectively [Table 1].

In general, the CMT1A and HNPP-affected patients harboring the I92V mutation manifest with CMT1A/HNPP 13 years earlier than the patients without I92V sequence variant.

The statistically significant association was not detected for the CMT1A/HNPP patients with early age at onset (before 10 years of age) harboring Ile92Val mutation.

We have also taken into consideration other features of the CMT1A/HNPP phenotype (lower and upper limb weakness, foot deformity requiring surgery, presence of deep tendon reflexes or sensory disturbances). No statistically significant associations have been found for clinical parameters of CMT1A/HNPP and the presence of I92V *LITAF* mutation.

To conclude, within numerous clinical parameters of CMT1A/HNPP phenotypes, only age at onset has been shown to be associated with the presence of I92V mutation in the group of older patients (after 10 years of age).

### The I92V LITAF sequence variant mislocalizes to the mitochondria

**Fig. 1:**
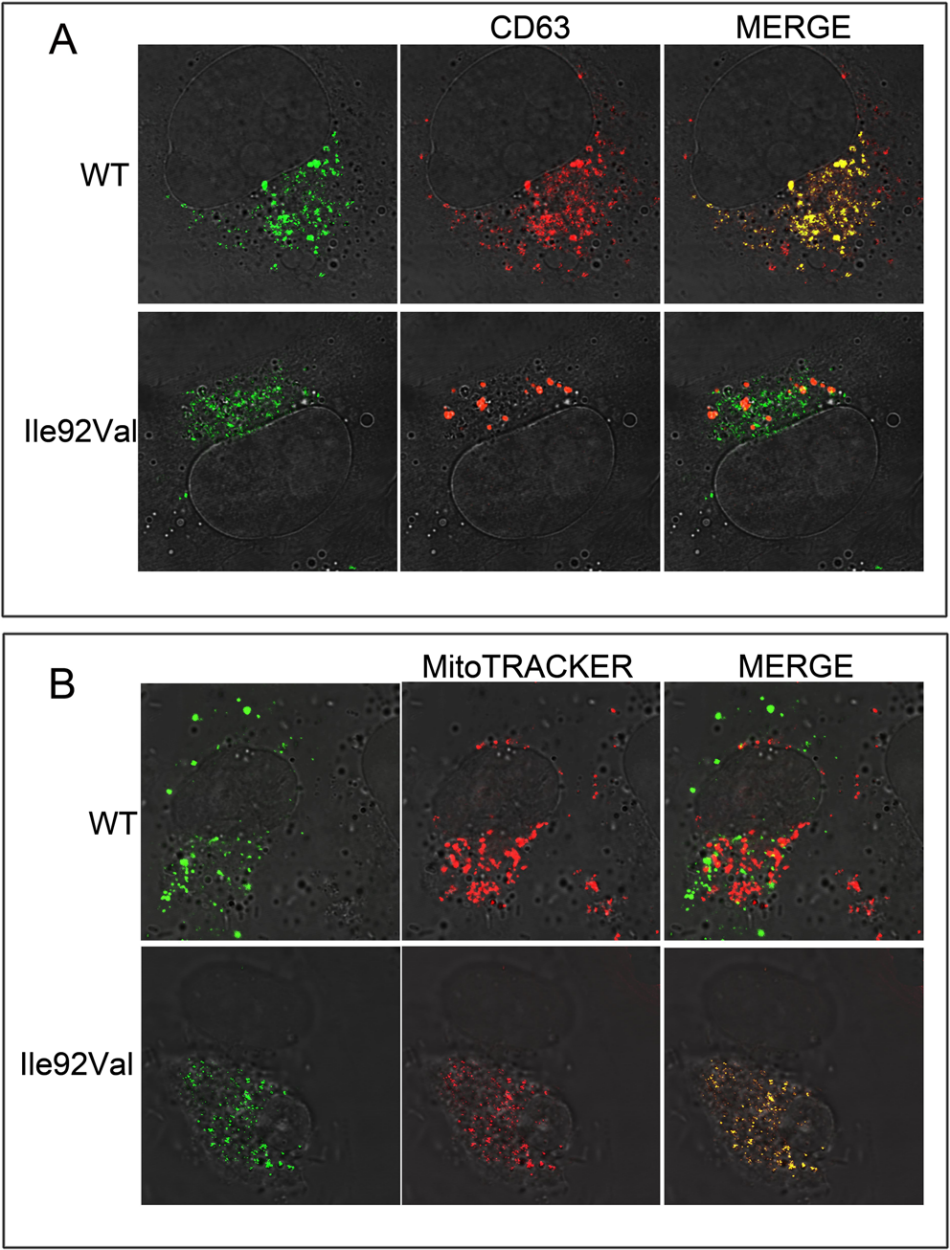
LITAF mutants show different location from WT LITAF. BGMK cells were transiently transfected with myc-tagged WT LITAF and Ile92Val. In 24-h post-transfection, the cells were **a** fixed and permeabilized before indirect immunofluorescence was used to detect LITAF using anti-myc antibody (*green*), and lysosomes were visualized using anti-CD63 (*red*) or **b** stained with MitoTRACKER (*red*) prior to the use of indirect immunofluorescence with anti-myc antibodies (*green*) to detect LITAF. Nuclei were visualized using differential interference contrast (DIC), and images were captured using a scanning confocal microscope

Since we observed differences in age of onset of CMT1A and HNPP in patients depending on the occurrence of the LITAF I92V sequence variant, we were interested to see if the I92V LITAF mutant is found in the same subcellular location as the WT LITAF. To further explore the relocation of LITAF, we transfected into BGMK cells either wild-type LITAF or I92V. We observed that WT LITAF co-localizes with the lysosomal marker CD63, suggesting that WT LITAF is normally present in the lysosomes (Fig. 1a). Next, we analyzed the localization of the I92V mutant and found that a portion of the I92V protein is localized to the lysosome (Fig. 1b) and a portion localizes with the mitochondria (Fig. 1b). This data suggests that a portion of the I92V mutant is mislocalized in the cell.

### LITAF effects protein levels of PMP22

**Fig. 2:**
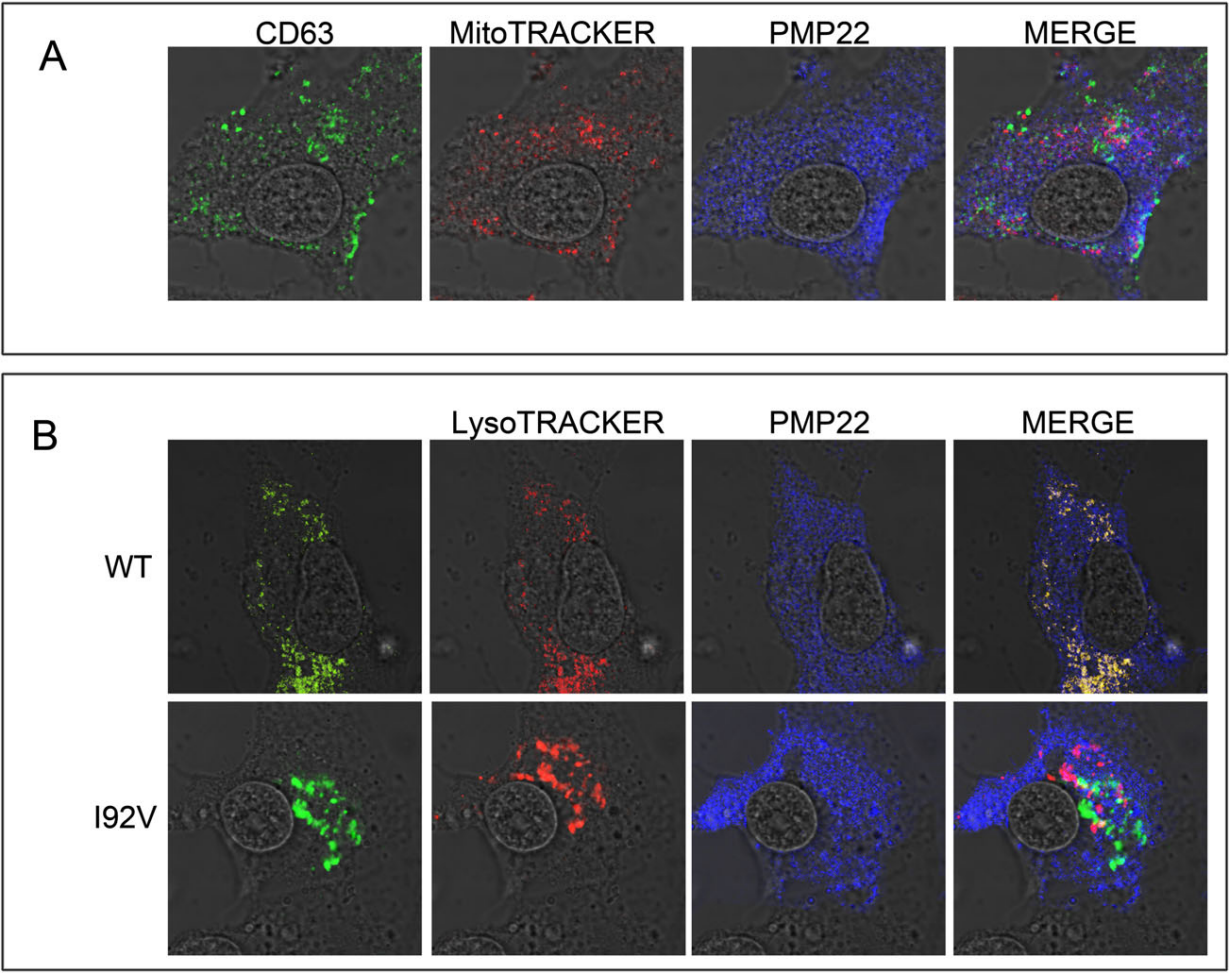
LITAF 192 V causes changes in the amount of PMP22 protein. BGMK cells were transiently transfected with **a**, **b** FLAG-tagged PMP22 and **b** myc-tagged I92V. LysoTracker or MitoTracker was applied to live cells 24 h post-infection, and cells were then fixed and permeabilized. Cells then underwent indirect immunofluorescence, and I92V was detected using anti-myc antibodies, and PMP22 was visualized using anti-FLAG antibodies. Nuclei were visualized using differential interference contract (DIC). All images were taken using a laser scanning confocal microscope

LITAF has been implicated in protein degradation [9, 23] while duplication of PMP22 results in CMT1A ([14], Lupski et al., 1991. Since mutations in LITAF and PMP22 produce a more severe form of CMT, we wondered if LITAF might be involved in regulating the amount of PMP22 protein. We co-transfected into BGMK cells either wild-type LITAF or I92V with flag-tag PMP22. We observed a 41 % increase in fluorescence when the cells were co-transfected with PMP22 and I92V compared to cells co-transfected with PMP22 and WT (Fig. 2), supporting the idea that LITAF may play a role in the degradation of PMP22.

## Discussion

In the present study, we show that the I92V sequence variant in addition to PMP22 mutations predispose patients to an earlier age of onset of both CMT1A/HNPP diseases. The I92V LITAF sequence variant has not been considered a biomarker of CMT1A/HNPP. Nevertheless, in a single CMT1A family study, the I92V sequence variant has been shown to contribute to a more severe CMT1A phenotype [12]. Similarly, Scelsa and co-workers reported an association between the I92V sequence variant and familiar inflammatory demyelinating polyneuropathy in a single family [17].

The I92V sequence variant occurs in different ethnic groups with a relatively high frequency in the healthy individuals. In our study, the I92V mutation was found in 47 % of CMT1A-affected patients and 54 % HNPP patients [Table 2]. Recently, in a small group of patients manifesting with early-onset HNPP, the I92V sequence variant within the *LITAF* gene was again found to be present [13]. Thus, CMT1A patients with an increased gene dosage of PMP22 and the I92V mutation may manifest CMT1A symptoms earlier than patients with a wild-type LITAF protein.

**Table 2:** The frequency of the Ile92Val substitution within *LITAF* gene in the patients and healthy subjects from different populations

Region	CMT1A (patients with duplication of *PMP22* gene)		HNPP (patients with deletion of *PMP22* gene)		CMT (patients without duplication or deletion of PMP22 gene)	Control group	Reference
USA	ND		ND		46 % (*n* = 192)	18 % (*n* = 94)	[16]
France	ND		ND		38 % (*n* = 35)	26 % (*n* = 50)	[8]
Denmark	ND		ND		na	16.2 %	[12]
Serbia	ND		ND		na	na (*n* = 75)	[6]
Poland	47 %		54 %		37.6 % (*n* = 49)	22 % (*n* = 50)	This study
	M (*n* = 38)	F (*n* = 31)	M (*n* = 27)	F (*n* = 10)			

*ND* not done, *na* data not available, *M* male, *F* female

Interestingly, a statistically significant effect of the I92V sequence variant on CMT1A/HNPP phenotype has only been found in the group of patients manifesting CMT1A/HNPP disease after 10 years of age. We therefore cannot preclude the action on the part of other modifying genetic factors in the case of a group of younger patients with a more severe, early age at onset clinical course for CMT1A/HNPP. In other words, *PMP22* gene dosage regulated by LITAF activity is not the single, exclusive molecular mechanism influencing the severity of CMT1A. While our study also analyzed other parameters of CMT1A, the only statistically significant result obtained was the correlation between age at onset and the I92V mutation within the LITAF gene.

Given the primary role of LITAF protein in the degradation of *PMP22*, the mislocalization of LITAF from lysosomes to mitochondria may be associated with a less effective degradation of *PMP22* protein. Since gene dosage is considered crucial to CMT1A severity, the decrease of LITAF within lysosomes may be a key factor in molecular pathogenesis of CMT1A. We therefore hypothesized that the *LITAF* gene might play a role in the regulation of *PMP22* gene dosage. In this study, we explored the effect of I92V LITAF on *PMP22* level within the cell and found through immunofluorescence that when BGMK cells are co-transfected with *PMP22* and I92V, there is a tendency for *PMP22* to accumulate in the cell, suggesting that LITAF plays a role on *PMP22* regulation.

In summary, our results indicate that the LITAF I92V sequence variant is associated with an earlier age of onset of the CMT1A/HNPP diseases and may be considered a genetic modifier of clinical variability. To conclude with respect to the Polish population at least, the I92V LITAF sequence variant may be considered a genetic modifying factor.
